# Ricin Trafficking in Cells

**DOI:** 10.3390/toxins7010049

**Published:** 2015-01-09

**Authors:** Robert A. Spooner, J. Michael Lord

**Affiliations:** School of Life Sciences, University of Warwick, Coventry CV4 7AL, UK

**Keywords:** ricin, ER-cytosol retrotranslocation, p24 proteins, ERP2, HRD1, proteasome, RPT, chaperone

## Abstract

The heterodimeric plant toxin ricin binds exposed galactosyls at the cell surface of target mammalian cells, and, following endocytosis, is transported in vesicular carriers to the endoplasmic reticulum (ER). Subsequently, the cell-binding B chain (RTB) and the catalytic A chain (RTA) are separated reductively, RTA embeds in the ER membrane and then retrotranslocates (or dislocates) across this membrane. The protein conducting channels used by RTA are usually regarded as part of the ER-associated protein degradation system (ERAD) that removes misfolded proteins from the ER for destruction by the cytosolic proteasomes. However, unlike ERAD substrates, cytosolic RTA avoids destruction and folds into a catalytic conformation that inactivates its target ribosomes. Protein synthesis ceases, and subsequently the cells die apoptotically. This raises questions about how this protein avoids the pathways that are normally sanctioned for ER-dislocating substrates. In this review we focus on the molecular events that occur with non-tagged ricin and its isolated subunits at the ER–cytosol interface. This focus reveals that intra-membrane interactions of RTA may control its fate, an area that warrants further investigation.

## 1. Introduction

**Figure 1:**
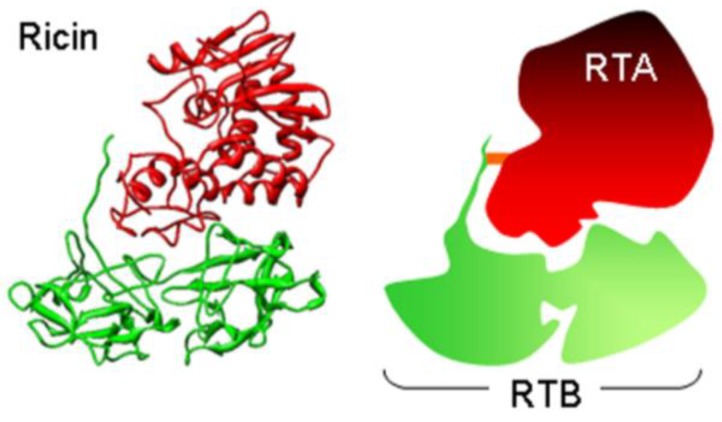
Crystal structure (left) and cartoon representation (right) of ricin holotoxin. The crystal structure (PDB code 2AAI [11]) was viewed in Chimera (UCSF) and is shown from the side with the receptor-binding surfaces of ricin toxin B chain (RTB, green) facing downwards. Ricin toxin A chain (RTA, red) and RTB are held together by hydrophobic interactions and a disulphide bond (orange).

Ricin is a toxic heterodimeric protein expressed in the seeds of the castor oil plant *Ricinus communis* L*.*, and comprises a cell binding B chain (RTB) disulfide-linked to a catalytic cytotoxic ribosome-inactivating protein (RTA, Figure 1). Cell-binding specificity of RTB on target mammalian cells is promiscuous and low affinity, depending on exposure of β1→4 linked galactose residues on multiple glycoproteins and glycolipids displayed at the mammalian cell surface [1,2,3]. Endocytosis of ricin is poorly understood. There is no obvious role for caveolae [4] in productive intracellular routing (that leading to intoxication) and cytotoxicity does not appear to follow from recruitment of ricin to receptors in coated pits, since inhibiting coated pit formation by hypotonic shock [5] or by acidification of the cytosol [6] makes little difference, even though there is a 50% decrease in ricin endocytosis. It is likely that the promiscuous binding of ricin results in multiple intracellular trafficking pathways following endocytosis, making the study of productive trafficking arduous. Receptors for cytotoxicity are likely to be proteins [1,7,8] and since high salt: low pH treatment sensitizes cells to ricin, misfolded cell-surface proteins may be productive receptors [9]. Other than this, no other definitive receptors have been identified, although a haploid cell gene-trap screen has identified a likely requirement for a cell-surface orphan G-protein coupled receptor (GPCR) family member, Gpr107 [10].

**Figure 2:**
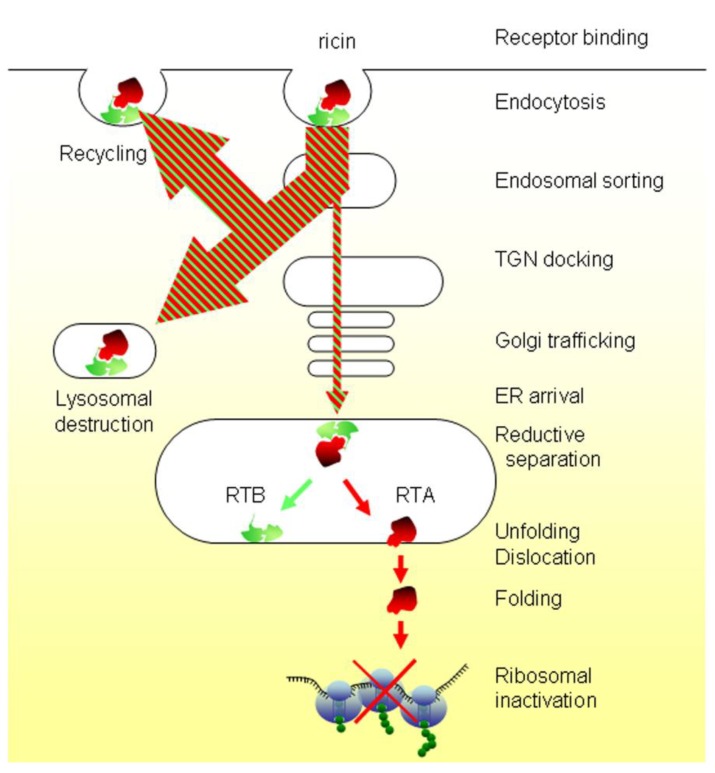
Intracellular trafficking of ricin. Ricin binds *N*-glycosylated molecules with available β1→4 linked galactosyls at the plasma membrane and after internalization by endocytosis, traffics via early endosomes, the TGN and the Golgi stack to the endoplasmic reticulum (ER), where reductive separation of the toxic ricin toxin A (RTA) from the cell-binding ricin toxin B (RTB) occurs. Free RTA retrotranslocates (dislocates), refolds in the cytosol and inhibits protein synthesis by catalytic removal of a key adenine residue on the 28S ribosomal subunit, at the site of EF-2 complex interaction [15]. Following a ribotoxic stress response and activation of multiple signaling pathways [16], ricin-treated cells die apoptotically [17].

To aid description of the productive pathway, reconstituted holotoxin comprising *C*-terminally tagged versions of RTA with plant-derived RTB have been employed. One of these tagged versions, RTA-sulf1, bears a motif that can be sulfated *in vivo* by the addition of [^35^S]O_4_^2−^ in a reaction catalyzed by the *trans*-Golgi cisternal resident tyrosylprotein sulfotransferases TPST1 and TPST2 [12], and so measures retrograde arrival of the toxin at the Golgi stack [13]: another, RTA-sulf2, bears the same sulfation signal and an additional set of motifs that can be *N*-glycosylated by oligosaccharyltransferase complexes upon arrival in the ER lumen [13]. RTA-sulf2-containing holotoxin has been used to demonstrate cytosolic delivery of a sulfated *N*-glycosylated RTA, thus defining a plasma membrane-Golgi-ER-cytosol retrograde trafficking route [14] (Figure 2).

Subsequently, roles for dynamin [18], cholesterol [19], calreticulin [20], PKA type IIα [21], Rab6A isoforms [22], sphingolipids [23], flotillin [24], and Derlin-1 [9] have been described for the retrograde transport of ricin reconstituted with sulf-tagged RTA variants. In addition, sulf-tagged RTA variants have been used to describe the productive intoxication pathway as clathrin-, Rab9- and Rab11-, and annexin-independent [25,26]. Only a small proportion of endocytosed holotoxin is transported in vesicle carriers via early/sorting endosomes and the Golgi complex to the endoplasmic reticulum (ER) lumen: the remainder is thought to enter both recycling endosomes and the destructive lysosomal pathway [27,28].

There may be a caveat: high concentrations of the small molecule reducing agent dithiothreitol (DTT) are required *in vitro* to reduce native ricin holotoxin to its constituent RTA and RTB chains [29,30], which suggests that the disulfide bond that bridges the C-terminal tail of RTA with RTB is normally occluded in the holotoxin. Comparison of the crystal structures of holotoxin and isolated RTA confirm that the *C*-terminus of free RTA protrudes, whilst in the holotoxin it is packed in a pocket on RTB that appears to be inaccessible [31]. Thus TPST-dependent sulfation of RTA-sulf1- and RTA-sulf2-containing holotoxins in the Golgi would presumably only occur if the junction between the two chains is destabilised by sulf-tagging, or if the sulfation motifs extrude from the RTA:RTB interface: either implies structural remodeling of the holotoxin at the junction of the two toxin subunits. It follows that the details of retrograde trafficking and molecular interactions of RTA revealed by the use of holotoxins containing *C*-terminally tagged RTA may reflect features of the tagged RTA chain and not necessarily that of native toxin.

In this review we therefore focus on the molecular events that have been elucidated with non-modified ricin and its isolated subunits (or confirmed with non-modified toxin) and confine ourselves to examining events at the ER–cytosol interface.

## 2. Er Luminal Events

Interaction of ricin with the ER-luminal chaperone protein disulfide isomerase (PDI) is thought to remodel the holotoxin structure to open the interface between RTA and RTB, thus permitting reductive cleavage [2,32,33]. Since PDI selects its substrates by binding permanently or transiently unfolded regions [34], and ricin holotoxin purified from seeds is thermally stable *in vitro* [35], then this suggests that ricin is either transiently unstable under ER conditions, or is destabilized in transit. This latter may provide a functional rationale for the proteolytic clipping of ricin that has been noted during passage through early endosomes [36,37]. Released RTA has a different conformation than RTA in holotoxin, with the displaced *C*-terminal tail exposing a relatively hydrophobic patch that had previously been occluded by RTB [31], and is thermally unstable, prone to aggregation at 37 °C [38]. This hydrophobic patch is a prime candidate for interactions with the ER Hsp70 family chaperone BiP which maintains the solubility of RTA in the ER lumen, thus protecting against cytotoxicity [39]: cytotoxicity follows from co-chaperone mediated release from BiP [40], possibly by transfer to the ER Hsp90 family chaperone GRP94 [38,41]. Although a number of compounds are thought to promote ER aggregation of RTA fusions [42], the extent of RTA aggregation in the ER lumen under normal conditions is unknown, as are any toxic effects that may be associated with this. In addition, interactions with the ER-enhancing mannosidase-like chaperone proteins EDEM1 and EDEM2 occur [43,44], although the mechanisms are not clear: the interactions appear to be independent of the lectin properties of the EDEMs, and are influenced by point mutations in the *C*-terminal hydrophobic patch of RTA [45].

Isolated RTA interacts via its hydrophobic patch (-KFSVYDVSIL**I**PIIALMVYR**C**A-) with lipid membranes, partially unfolding in the process, with direct evidence that the emboldened residues I249 and C259 enter the non-polar hydrophobic core of the membrane [46]. This C-terminal hydrophobic region has features typical of a single-spanning *trans*-membrane domain, and can be recognized as such by the predictive topology programs TOPCONS (http://topcons.cbr.su.se/) [47] and TOPCONS-single (http://single.topcons.net/) [48]. We have suggested that membrane insertion may initiate retrotranslocation (dislocation) from the ER, with RTA masquerading as a misfolded membrane protein that is removed to the cytosol by the ER quality control ERAD (ER protein degradation) system [31]. However, whilst ERAD substrates are dispatched by cytosolic proteasomes [49], RTA is not.

## 3. ER–Golgi Cycling Precedes Dislocation

RTA can be directed into the ER lumen of the yeast *Saccharomyces cerevisiae* by expressing a recombinant version with an *N*-terminal signal peptide [50,51] which is removed during ER entry, so that the ER-directed RTA is non-tagged [40]. This approach should allow ER-cytosol events to be examined independently of any confounding plasma-membrane to ER trafficking effects, but we note that ricin holotoxin interactions in the ER cannot be mapped in this way: for example, the reductive cleavage that separates RTA and RTB. The genetic tractability of yeast, its ability to survive deletions of functions that are essential in mammalian cells and the availability of knock-out libraries allowed us to examine dislocation in yeast mutants lacking known components of ERAD quality control systems [40]. Judicious selection of RTA mutants attenuated in catalytic activity permits correlation of yeast growth (a proxy for cytotoxicity) with RTA stability assays: thus deletion of a function required for cytosolic entry of RTA permits growth with RTA trapped stably in the ER, whilst deletion mutants that permit cytosolic entry of RTA grow slowly with RTA being turned over in the cytosol.

These studies revealed a role for coat protein complex II (COPII)-mediated transport to the Golgi prior to ER return and dislocation. Formation of the multi-subunit COPII coat at specialized ER-exit sites is coupled with cargo packaging as a prelude for cargo transport to the ER—Golgi intermediate compartment [52]. Coupling of cargo to coat formation is achieved in part by *trans*-membrane cargo receptors that interact with the COPII coat complex. COPII-mediated ER-Golgi cycling of ERAD substrates prior to dislocation and destruction is well established [53,54]. However, the yeast RTA expression studies revealed an unexpected, very strong deletion phenotype associated with this cycling: Δ*erp2* yeast grew well when expressing attenuated RTA, and also survived expression of fully active RTA [40].

The yeast p24 proteins (Erp1p-6p, Emp24p and Erv25p) are functionally non-redundant Type I transmembrane proteins predominately localized to Golgi and ER membranes, with ill-defined roles in cargo selection and cycling between these compartments [55,56]. Each is non-essential for growth and deletion phenotypes are typically minor. Thus, deletion of *ERP1* or *ERP2* causes a delay in maturation of the glycosylphosphatidylinositol (GPI)-linked Gas1p, deletion of *EMP24* or *ERV25* delays secretion of invertase and maturation of Gas1p, simultaneous deletion of these four genes causes no additional effects over that of single deletions on protein secretion or growth and deletion of any p24 gene results in increased secretion of Kar2p, the yeast BiP homolgue [57,58]. Indeed, all eight p24 genes can be deleted without loss of viability [59].

**Figure 3:**
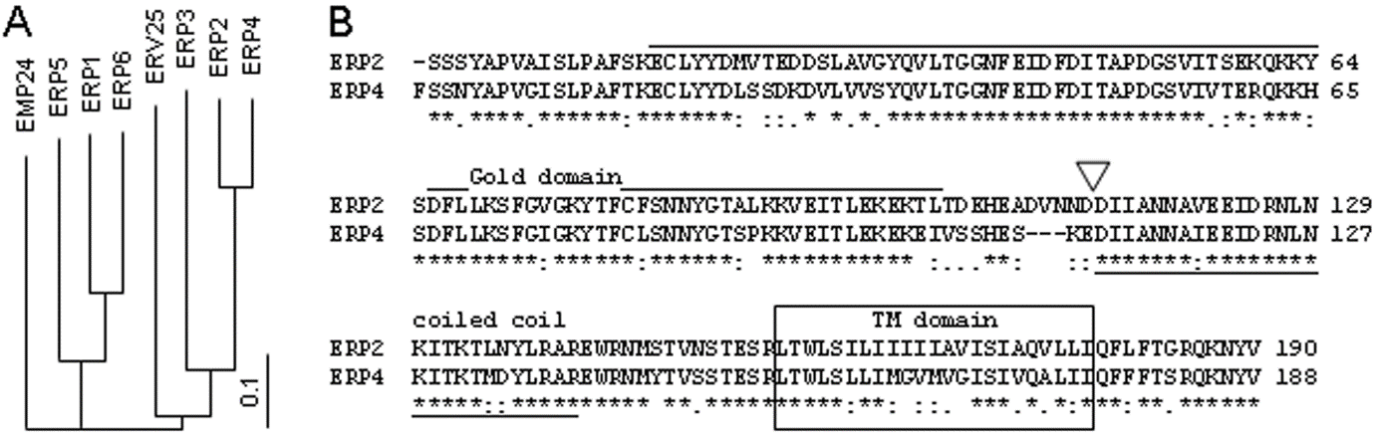
Comparison of *S. cerevisiae* p24 proteins. (**A**): Amino acid comparisons were made in ClustalW [60], and are shown as a tree prepared in TREEVIEW [61]; (**B**): The striking similarity of Erp2 and Erp4 proteins. Overlined, GOLD domain; underlined, coiled-coil domain; boxed, *trans*-membrane domain; amino-acid similarity (*:.), identity, conserved, and less-conserved respectively; white triangle, junction of gene fusions between the GOLD and coiled-coil domains.

The very strong requirement [40] for Erp2p for RTA intoxication is surprising since deletion of individual p24 proteins perturbs the expression of others, and Erp2p and Erp4p are highly similar (Figure 3) and functionally redundant when measured by Kar2p secretion [57].

**Figure 4:**
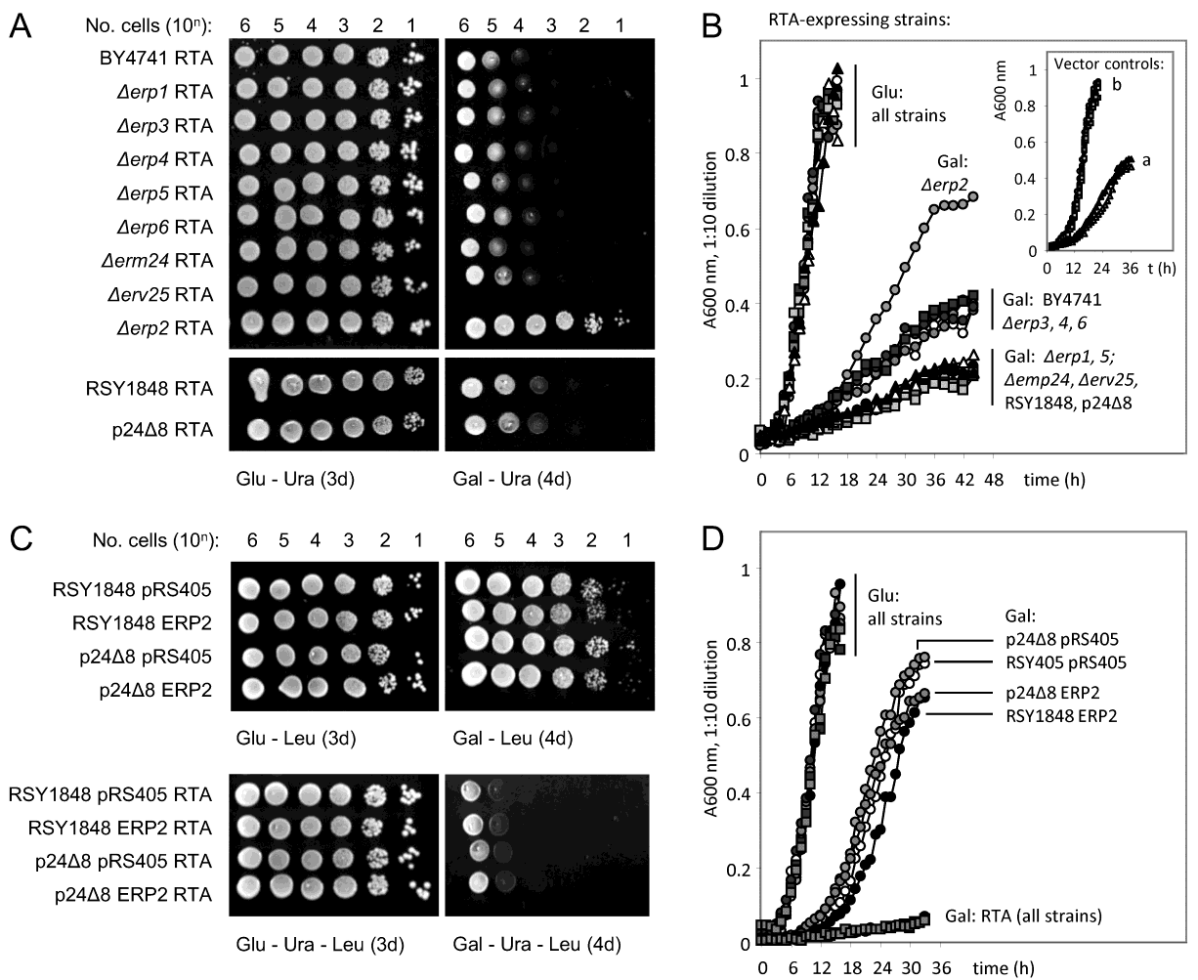
Requirement of p24 proteins for toxicity of RTA in *S. cerevisiae*. (**A**): Upper panels: growth of wt (BY4741) and p24 deletion strains (upper panels) transformed with an expression plasmid (URA selection) for ER-targeted RTA on agar containing glucose (glc, repressing, left-hand panels, 3d growth) and galactose (gal, inducing, right-hand panels, 4d growth). Lower panels; growth of wt (RSY1848) and its deletion derivative (p24Δ8) lacking all eight p24 genes, otherwise as in (**A**,**B**) Growth of these strains in liquid medium. Circles: white, BY4741; increasing tone, *Δerp1*, *Δerp2*, *Δerp3*, *Δerp4*, and *Δerp5*, respectively. Squares: black, *Δerp6*; dark grey, *Δemp24*; mid-grey, *Δerv25.* Triangles: white, RSY1848; black, p24Δ8. Inset: growth of these strains transformed with vector on galactose. a, RSY1484, p24Δ8; b, all other strains. Symbols as in main panel. (**C**): Upper panels: growth of wt (RSY1484) and p24Δ8 yeast transformed with an expression vector for ERP2 (LEU selection) or its vector control (pRS405, LEU selection) on glucose (**left**) and galactose (**right**) agar. Lower panels: growth of the strains from the upper panels, all transformed with an expression vector for RTA. (**D**): Growth of all strains from C in liquid selective medium containing glucose or galactose.

Spotting dilutions of yeast cells with inducible expression of ER-targeted RTA onto glucose (non-inducing) and galactose (inducing) selective media (Figure 4A) confirmed this normal requirement of Erp2p expression for cytotoxicity of RTA. Good growth on galactose was apparent in the *Δerp2* strain only, but not in the other p24 single deletion strains nor in a strain (p24Δ8) in which all eight p24 genes are deleted. Growth in selective liquid media (Figure 4B) revealed a further subtlety, where single deletion of *ERP1*, *ERP5*, *EMP24* or *ERV25* sensitized yeast to the effects of RTA expression. Thus Erp1p, Erp5p, Emp24p and Erv25p appear normally to inhibit engagement of ER-localized RTA with components of the COPII-mediated transport process that permits access to the Golgi. There is no discernible role for the products of *ERP3*, *ERP4* or *ERP6* since single deletions of these genes had no measurable effect on growth of cells expressing RTA (compare with wt). The p24Δ8 deletion (that lacks all p24 proteins) expressing RTA grew at the same rate as its parent RSY1848 similarly expressing RTA. These results are consistent with a model where individual p24 proteins act as specific receptors that allow access of cargo to COPII components, whilst the others preclude entry to COPII buds: but in the absence of all p24 proteins, access to these sites is unhindered [59].

Stable overexpression of Erp2p (Leu selection) in a strain that otherwise expresses all eight p24 proteins (RSY1848) did not sensitize the cells further to inducible RTA expression compared to cells stably transformed with the parental plasmid (pRS405, Figure 4C,D). Similarly, we could measure no obvious effect of stable sole expression of Erp2p in the p24Δ8 strain (Figure 4C,D): the only obvious effect is that Erp2p expression in both contexts results in slightly reduced growth rates in galactose medium (Figure 4D). We conclude that the effects of Erp2p on RTA cytotoxicity can only be measured clearly in the context of expression of the other p24 proteins.

**Figure 5:**
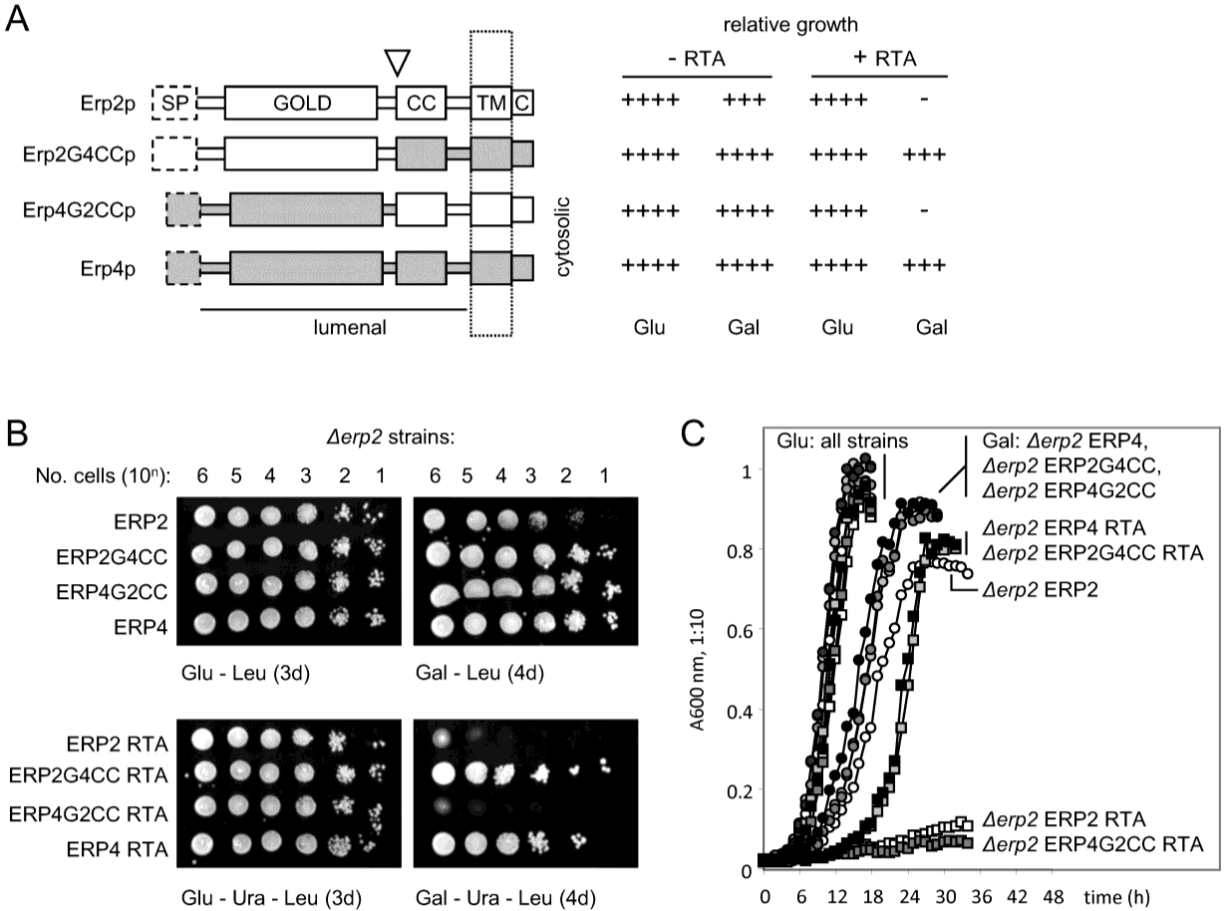
Overexpression of ERP2 and ERP2-ERP4 gene fusions. (**A**): Structures of the ERp2p and ERp4p proteins and the predicted structures of the reciprocal hybrids following domain swap (Epp2G4CCp and Erp4G2CCp), and their relative growth (determined from (**B**)). SP, signal peptide (dashed boxes); GOLD, luminal GOLD domain; CC, coiled-coli domain; TM, *trans*-membrane domain; C, cytosolic domain, dotted box, ER membrane, white triangle, junction of the GOLD-CC domain swap; (**B**): Upper panels: growth of the *Δerp2* strain transformed separately with expression vectors for ERP2, ERP4 and the reciprocal hybrids (all LEU selection) on glucose and galactose agar. Lower panels: the same strains subsequently transformed with an expression vector for ER-targeted RTA (URA selection). C. Growth of the strains in (**B**) in liquid selective media.

The p24 proteins have a conserved architecture (Figure 5A), with an N-terminal GOLD (Golgi dynamics) domain defined bioinformatically [62], a predicted coiled-coil (CC) domain thought to be involved in p24 multimerisation [63], a *trans*-membrane domain (TM) and a short cytosolic tail (C) that interacts with components of the COPII and COPI coatamer machineries [64]. Since stable expression of ERP2 in the *Δerp2* strain restored RTA cytotoxicity (Figure 5B,C), confirming our previous results [40], but overexpression of ERP4 in the *Δerp2* strain did not (Figure 5B,C), even though Erp2p and Erp4p are highly similar (Figure 3), then the differences in behavior between Erp2p and Erp4p depend on relatively few amino acids. To examine this we constructed and expressed Erp2p-Erp4p chimeras, expressed them stably in the *Δerp2* strain and then tested these derivatives after RTA expression.

Reciprocal gene fusions were made at the GOLD/CC domain boundaries (white triangle, Figure 3B and Figure 5A). Expression of a fusion with the GOLD domain of Erp2p and the remaining domains of Erp4p (Erp2G4CCp) in a *Δerp2* strain did not restore toxicity when RTA was expressed (Figure 5B,C). However, the reciprocal fusion with the GOLD domain of Erp4p and the remaining domains of Erp2p (Erp4G2CCp) was, like expression of Erp2p, able to complement the Erp2p deficiency when assayed by growth during RTA expression.

**Figure 6:**
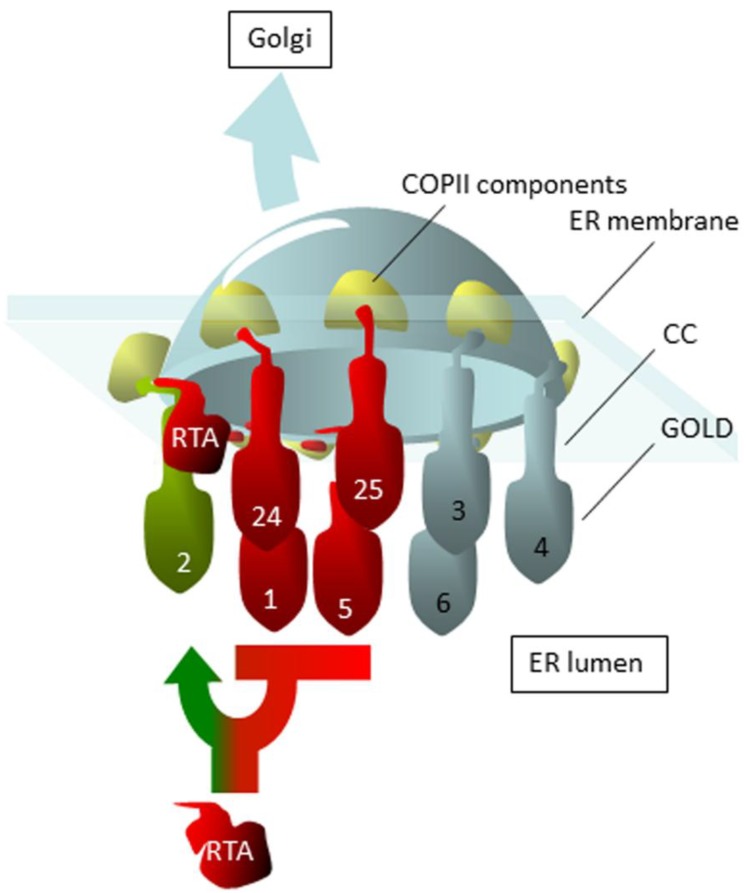
Proposed cycling of ricin toxins A (RTA) chain between the endoplasmic reticulum (ER) and Golgi. Erp1p, Erp5p, Emp24p and Erv25p (1, 5, 24, 25 respectively) prevent entry into COPII coated ER membrane buds, and reduce toxicity: Erp3p, Erp4p and Erp6p play little or no role (3, 4 and 6 respectively). In contrast, in the presence of other p24 proteins, dislocation of RTA is an Erp2p (2)-dependent process with key roles close to or within the membrane. In the absence of all p24 proteins, ER-targeted RTA is toxic to yeast, suggesting unfettered access to the budding vesicle. GOLD, CC: GOLD and coiled-coil domains, respectively.

Thus, despite suggestions that the GOLD domain is a cargo receptor [56,65], we find no evidence that the nature of the GOLD domains of Erp2p and Erp4p determines the toxicity of ER-expressed RTA. The domain-swap experiments suggest that the important Erp2p sequences for interacting with RTA lie very near to, or within, the ER membrane (Figure 6). Following the outcome of these interactions, a molecular decision is made, leading to a choice of vesicular transport or dislocation: the nature of this decision is influenced by the *N*-glycosylation status of RTA [66].

Ricin intoxication of mammalian cells is sensitive to perturbations of complexes mediating anterograde and retrograde transport between the ER and the Golgi [67]. Roles for COPII components and an Erp2p homologue have also been noted in a siRNA screen of *Drosophila* cells, suggesting that ER-Golgi cycling is a universal feature of ricin intoxication [68]. In the light of the yeast studies, it may be that these findings really reflect cycling of free RTA rather than of holotoxin.

## 4. ER-Cytosol Dislocation

Following p24 interactions, RTA dislocates to the cytosol. The dislocon has core functions that can be adapted for a range of ER substrates in a “bolt-on” manner [69]: when tested in the yeast system, RTA dislocation requires few of these, with minimal roles for the core Hrd1p dislocon and its regulator Hrd3p, and an intermediate requirement for Der1p, an adaptor for luminal protein substrates [40]. Similarly, in mammalian cells, direct ER expression of an attenuated RTA reveals a dislocation role for Sel1L, the mammalian homologue of the yeast Hrd3p [70]. In the yeast system, RTA requires a particular amino acid (L74) of Hrd1p [71]. Curiously, this residue lies in a cytosolic loop of Hrd1p and is specific for membrane-embedded (ERAD-M) substrates [72]—a strong suggestion that the embedded *C*-terminal tail of RTA protrudes through the membrane and makes cytosolic interactions with Hrd1p.

An early suggestion was that the high arginyl:lysyl bias of RTA (it has only two lysyl residues) allowed dislocation to occur by an ERAD-like process, with escape from destruction afforded by lack of suitable sites for ubiquitylation [73]. Dislocation of luminal and membrane-bound ERAD substrates typically requires polyubiquitylation of the substrate on the cytosolic face by dislocon-dedicated E3 ligases: in the yeast system, this is supplied by the RING domain of Hrd1p [74], and in mammals by the RING domains of HRD1 [75]. However, neither mutational inactivation of the Hrd1p E3 ligase activity nor replacement of the two RTA lysyl residues with arginyls affects the dislocation of RTA: thus its dislocation is independent of ubiquitylation [40].

The extraction motor for ERAD substrates is typically a complex of the hexameric AAA-ATPase cdc48p (yeast)/p97 (mammals) with ubiquitin-handling co-factors [76], in a process that may involve deubiquitylation and reubiquitylation for subsequent proteasomal targeting [77,78]. Avoidance of dislocation-associated ubiquitylation allows RTA to dislocate in a cdc48-independent manner: instead RTA utilizes the AAA-ATPase activity of the proteasomal Rpt4p subunit [40], which can also act as an ER extraction motor [79].

## 5. Cytosolic Triage

**Figure 7:**
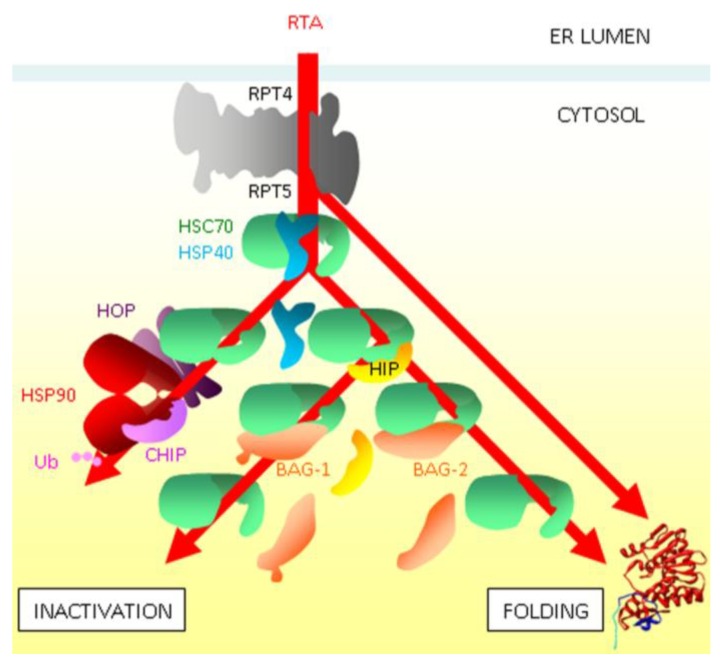
Post-dislocation scrutiny by a network of chaperones determines the cytosolic fate of RTA [38,40,81]. Following extraction from the ER by the RPT4 subunit of the proteasome, interactions with RPT5 allow RTA to recover activity. The role of Hsc70/Hsp40 interactions may be continuous scrutiny of RTA, preventing aggregation, and inactivation and activation (folding) fates follow from release of RTA from this complex. Transfer to Hsp90 via the Hsc70-Hsp90 operating protein HOP leads to CHIP-mediated ubiquitylation (Ub, ubiquitin) and inactivation of RTA. The Hsc70-interacting protein HIP stabilizes the Hsc70:RTA complex, and subsequent release by the BAG family guanine nucleotide exchange factors BAG-1 and BAG-2 leads to inactivation or folding respectively.

The findings that RTA and other ER-targeting toxic A chains have a low lysyl content led to a model where reduced opportunities for ubiquitylation allowed a proportion of dislocated catalytic A chains to avoid proteasomal destruction [73], and reports that pharmacological inhibition of proteasomal proteolytic activity sensitized mammalian cells to ricin were interpreted in this light [14,80]. However, dislocation studies in yeast revealed no obvious role for proteasomal proteolysis [40], and subsequent reinvestigation of this in mammalian cells revealed that the apparent sensitization of cells to ricin by pharmacological inhibition of proteasomal proteolysis simply reflects a time-dependent toxicity of proteasome inhibition [81]. An early event appears to be transfer of RTA from RTP4 to its neighbouring AAA-ATPase RPT5 subunit (Figure 7). Following this, RTA can recover catalytic activity, both *in vitro* and *in vivo* [81]: thus the RPT5 subunit has chaperone functions. In addition there are interactions with the cytosolic chaperone Hsc70 [38]: the final fate of Hsc70-bound RTA depends on the prevailing concentration of co-chaperones that govern the competing processes of ubiquitylation and productive folding. Currently, mechanisms for inactivation of RTA in the cytosol are unclear: HOP-mediated transfer from Hsc70 to Hsp90-CHIP E3 ligase complexes or BAG-1 mediated release might both direct RTA to the proteasome (the former by ubiquitylation and the latter via the BAG-1 interlaced ubiquitin-like domain), yet proteasomal proteolysis of RTA is not seen [81]. It may be that the aggregation-prone RTA is under continual RPT5 and Hsc70-mediated scrutiny, with continual attrition of the cytosolic RTA population from failure to maintain a stable folded soluble state.

## 6. Concluding Remarks

Ricin cytotoxicity to mammalian cells is multifactorial, depending on the availability of productive receptors at the cell surface, successful traffic through (likely multiple) ER targeting pathways, interactions of holotoxin and reductively separated toxin subunits with ER chaperones, dislocation of RTA, and negotiation of this subunit through a complex network of cytosolic chaperones. Ultimately, a sufficient proportion of RTA refolds in the cytosol and inhibits protein synthesis by catalytic removal of a key adenine residue on the 28S ribosomal subunit [15]. A ribotoxic stress response follows, with activation of multiple signaling pathways [16], and eventually ricin-treated cells engage with apoptotic programs and die [17]. In many cases, experimental design can allow separation of these multiple events: for example, time-courses of intoxication in mammalian cells to separate likely trafficking effects from later events and the use of the yeast ER-targeting approach to allow focus away from ER-trafficking, leading to the description of complex interactions of RTA at or near the ER-cytosol interface. Although most of the molecular details have been uncovered using yeast studies, these should provide clues for mammalian studies which are complicated by the potent cytotoxicity of ricin: it is encouraging that Sel1L, the mammalian homologue of the yeast Hrd3p, was identified as a regulator of ricin A chain toxicity in mammalian cells [70], and the roles of the proteasome in RTA interactions in both systems appear to be equivalent [81].

We note that there are two similar examinations of RTA by ER and cytosolic chaperones, with Hsp70 family members maintaining RTA solubility so that cytotoxicity follows from co-chaperone mediated release. However, events preceding these interactions differ, with PDI interactions and proteasome interactions in the ER and cytosol respectively. Free RTA utilizes ERAD components for dislocation, but uncouples from the final steps of ERAD by dislocating in an ubiquitin-independent manner, thereby avoiding cdc48-complex interaction and subsequent proteasomal degradation. Upon entry to the cytosol, RTA is not degraded by the proteasome, instead interacting with a network of molecular chaperones and co-chaperones that includes the proteasome regulatory particle itself [38,81], thereby allowing a proportion of dislocated RTA to adopt a catalytic conformation. The ability of the proteasome to chaperone RTA suggests a failsafe mechanism for protein turnover in the cytosol, with the proteasome able to parse substrates and make decisions about their ultimate fate: the proteasome is judge, jury and executioner.

Focusing on membrane-associated events leads us to a speculative view: the membrane embedding of the RTA C-terminus may be more important than previously recognized. A simple domain-swap that interchanges the GOLD domains of Erp2p and the closely related Erp4p reveals that Erp2p interactions with RTA likely occur within the ER membrane; the interactions of RTA with a particular cytosolic loop of the dislocon-integral Hrd1p suggests that the C-terminus of RTA pierces the ER membrane, allowing it to interact with a *trans*-membrane segment of Hrd1p whose only known role is to interact with membrane proteins [71]; and, curiously, a single point mutation (P250A) in the membrane-embedded *C*-terminus of RTA reduces the role of EDEMs in RTA dislocation [44,45]. Since the EDEM interactions with RTA appear to be independent of their lectin activity and the region of RTA flanking P250 inserts into the ER membrane as a prelude to cytotoxicity [46], we speculate that the interaction with EDEMs may be an intra-membrane event, suggesting a role for EDEMs as intra-membrane sorting agents for some ERAD substrates.
